# Dopamine signaling: target in glioblastoma

**DOI:** 10.18632/oncotarget.1835

**Published:** 2014-03-20

**Authors:** Jiri Bartek, Zdenek Hodny

**Affiliations:** Danish Cancer Society Research Center, Copenhagen, Denmark; and Department of Genome Integrity, Institute of Molecular Genetics, v.v.i., Academy of Sciences of the Czech Republic, Prague, Czech Republic; Department of Genome Integrity, Institute of Molecular Genetics, v.v.i., Academy of Sciences of the Czech Republic, Prague, Czech Republic

Despite numerous promising discoveries in contemporary cancer research, and the emerging innovative cancer treatment strategies, the global burden of malignant diseases is expected to rise, in part due to the rapidly aging human populations. Consequently, there is an urgent need to design and validate new treatments in oncology, especially for cancer types with presently dismal prognosis due to limited treatment options, such as for patients suffering from glioblastoma multiforme. The efforts to address such need rely in large part on the recently introduced powerful technologies including high-throughput screens to uncover genetic and functional vulnerabilities of specific types of human tumors. Such effort also raises hopes to identify molecular targets so far associated with pathologies other than malignancies, and thereby bring about opportunities to repurpose already existing, approved drugs that target such mechanisms. Apart from lower cost of introducing such treatments for oncological diseases, drug repurposing is also good news due to much shorter time needed from target discovery to clinical applications, an aspect particularly valuable in disease contexts such as glioblastoma.

A successful example of the latter approach has now been reported in a new exciting study published in Oncotarget. Through a series of genome-wide shRNA screens, Li et al. [1] globally defined the set of human genes required for glioblastoma growth *in vitro*. The screen design differed from previous genome-wide shRNA screens in its subtractive nature, where gene silencings that caused lethality in lung cancer cell lines were “subtracted” from those causing lethality in glioblastoma lines. In this way, the authors identified genes specifically required for glioblastoma growth. Analysis of the gene list revealed a rather unexpected discovery – the top five pathways represented in this gene list involved neuro-transmitter receptor signaling. The authors subsequently demonstrated that one of these pathways, mediated by the dopamine receptor subtype 2 (DRD2), plays a critical role in glioblastoma mitogenic signaling.

**Figure 1:**
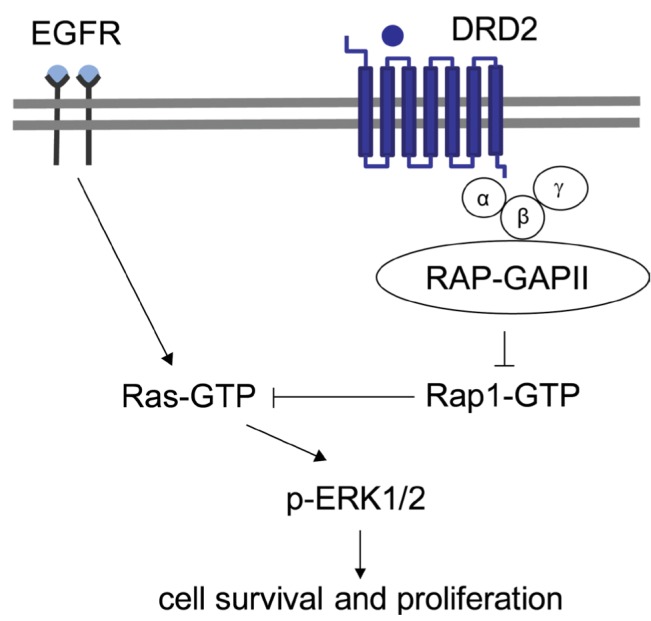
Targeting synergistic dependency on MAPK and dopamine signaling in glioblastoma Canonical pro-survival and mitogenic MAPK signaling, commonly deregulated in glioblastoma, is depicted as a simplified signaling cascade of EGFR, Ras-GTP and MAPK kinases Erk1 and Erk2 (Erk1/2). At the Ras node, MAPK pathway is positively modulated by dopamine signaling. Upon ligand binding, the DRD2 receptor holds inactive small GTPase Rap1 through activation of heterotrimeric G protein alpha i2 (alpha/beta/gamma) and the GTPase-activating protein RAP (RAP-GAPII), which promote GDP-bound (inactive) Rap1. Rap1 is a Ras antagonist and its inactivation thus results in amplification of MAPK signaling. Therefore, combined targeting of both pathways can offer a promising strategy for glioblastoma therapy.

Upon activation, DRD2 activates the trimeric G-protein complex through a canonical interaction. The G subunit of this complex, in turn recruits a GTPase that hydrolyzes RAP1-GTP, a small G-protein in the Ras superfamily [2]. In contrast to Ras-GTP, which binds/ activates Raf-1 and initiates the canonical mitogenic MEK/ERK signaling cascade, RAP1-GTP binds to but does not activate Raf-1 [2]. The hydrolysis of RAP1-GTP releases Raf-1 from sequestration, thereby allowing MEK/ ERK signaling (Figure 1).

Importantly, the authors demonstrated that DRD2 antagonists, clinically used as anti-psychotic drugs, harbor anti-glioblastoma activities. Moreover, these activities are synergistic when combined with EGFR inhibition. As discussed in the article, the work offers significant translational implications and suggests that FDA approved anti-psychotic agents may be repurposed as glioblastoma therapeutics. The notion is particularly attractive since these agents are known to cross the blood-brain barrier.

Beyond the obvious translational implications of dopamine antagonists as glioblastoma therapeutics, the findings by Li et al. raise additional biological implications. First, it is well-appreciated that glioblastoma is an aggressively infiltrative disease. Yet, it rarely metastasizes beyond the central nervous system (CNS) [3]. The observation that neurotransmitters, such as dopamine, are required for glioblastoma growth provides one explanation for this unusual phenotype. The non- CNS microenvironment may not possess the level of neurotransmitters required to sustain glioblastoma growth.

Another puzzle in glioblastoma therapeutic development involved the observation that while Epidermal Growth Factor Receptor (EGFR) dysregulation is critically important in the pathogenesis of glioblastomas [4], EGFR inhibitors are clinically ineffective [5]. The cross-signaling between dopamine receptor and EGFR offers an explanation for the poor clinical efficacy of inhibitors of receptor tyrosine kinases such as EGFR. It is likely that during glioblastoma pathogenesis, EGFR re-wired the molecular circuitry of the astrocyte as to “hijack” the neurotransmitter-mitogenic signaling axis [6]. Thereafter, the high concentrations of neurotransmitter in the CNS signal to effector molecules downstream of EGFR, thereby bypassing the need for EGFR activation.

To the extent that dopamine influences reward systems in the brain, emotion, and personality traits, the work further raises the possibility that personality and emotion may impact the risk of cancer development or growth through modulation of dopamine release. Interestingly, studies have related cancer risks to personality types [7].

There is an emerging recognition of the role of neurotransmitters in regulating cancer phenotypes. For instance, Magnon et al. published in a recent Science article that prostate cancers are infiltrated with parasympathetic cholinergic fibers. Moreover, the cholinergic neurotransmitters released by these fibers promoted tumor dissemination [8]. The work done by Li et al. [1] adds to this growing literature and highlights the importance of the nerve elements in the tumor microenvironment. In this context, drugs that modulate neuro-transmitter function warrant consideration as anti- neoplastic agents.
